# Supplementary figures and images for: Genome-Wide Characterization of Four Gastropod Species Ionotropic Receptors Reveals Diet-Linked Evolutionary Patterns of Functional Divergence
Source: Animals (Basel). 2026 Jan 7;16(2):172. doi: 10.3390/ani16020172 (PMC12837371; doi:10.3390/ani16020172)

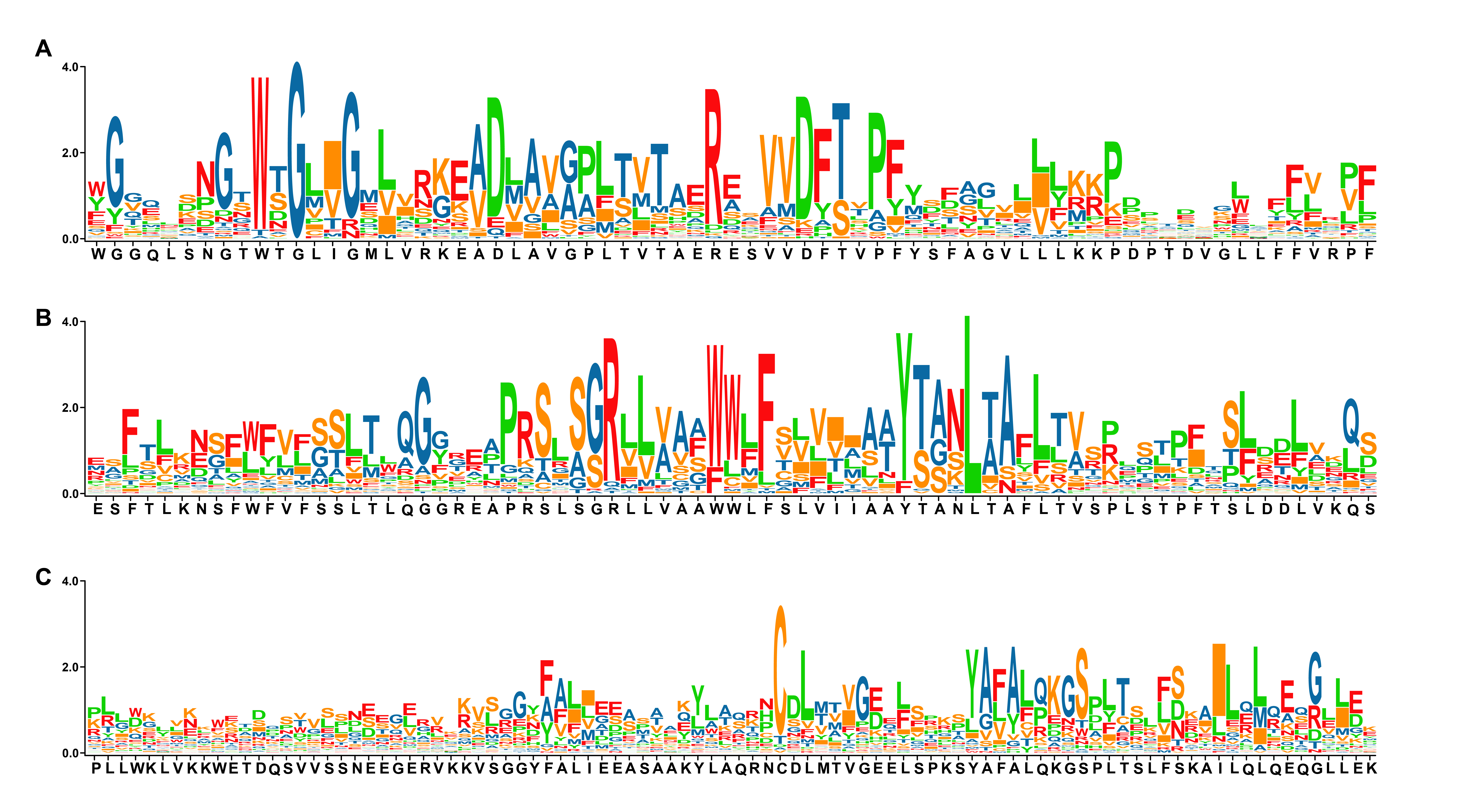

Supplement: Supplementary file 1 [file animals-16-00172-s001.zip › Figure S1.jpg]

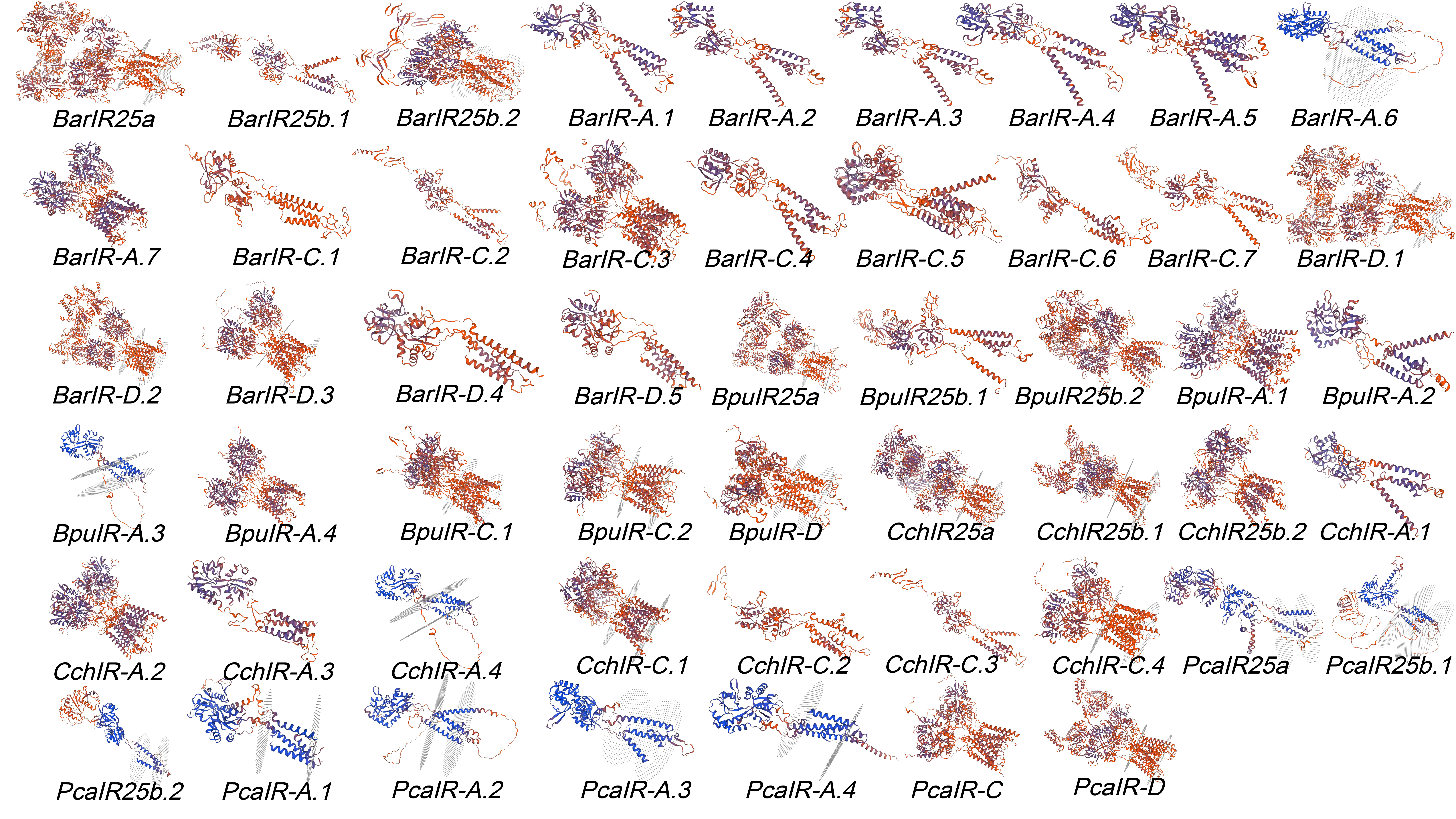

Supplement: Supplementary file 1 [file animals-16-00172-s001.zip › Figure S2.jpg]

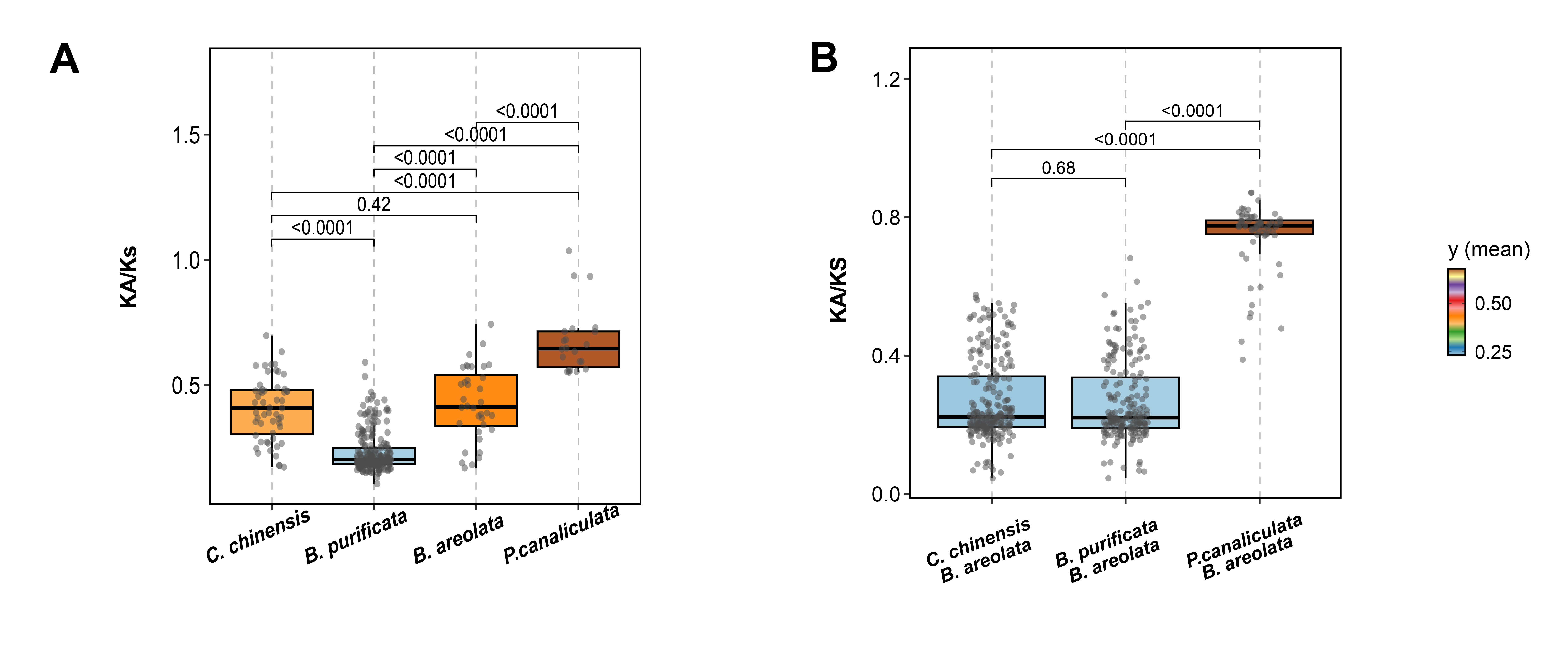

Supplement: Supplementary file 1 [file animals-16-00172-s001.zip › Figure S3.jpg]
